# Ternary Nanohybrid of Ni_3_S_2_/CoMoS_4_/MnO_2_ on Nickel Foam for Aqueous and Solid-State High-Performance Supercapacitors

**DOI:** 10.3390/nano12111945

**Published:** 2022-06-06

**Authors:** Sumanta Sahoo, Ganesh Dhakal, Woo Kyoung Kim, Jae-Jin Shim

**Affiliations:** School of Chemical Engineering, Yeungnam University, 280 Daehak-Ro, Gyeongsan 38541, Gyeongbuk, Korea; sumanta95@gmail.com (S.S.); gdhakal17@gmail.com (G.D.); wkim@ynu.ac.kr (W.K.K.)

**Keywords:** supercapacitor, CoMoS_4_, MnO_2_, Ni_3_S_2_, binder-free, nanohybrid

## Abstract

To overcome the issues related to supercapacitor (SC) electrodes, such as high cost, low specific capacitance (*C_s_*), low energy density (ED), requirements for expensive binder, etc., binderless electrodes are highly desirable. Here, a new ternary nanohybrid is presented as a binder-free SC electrode based on Ni_3_S_2_, CoMoS_4_, and MnO_2_. A facile two-step hydrothermal route, followed by a short thermal annealing process, is developed to grow amorphous polyhedral structured CoMoS_4_ and further wrap MnO_2_ nanowires on Ni foam. This rationally designed binder-free electrode exhibited the highest *C_s_* of 2021 F g^−1^ (specific capacity of 883.8 C g^−1^ or 245.5 mAh g^−1^) at a current density of 1 A g^−1^ in 1 M KOH electrolyte with a highly porous surface morphology. This electrode material exhibited excellent cycling stability (90% capacitance retention after 4000 cycles) due to the synergistic contribution of individual components and advanced surface properties. Furthermore, an aqueous binder-free asymmetric SC based on this ternary composite exhibited an ED of 20.7 Wh kg^−1^, whereas a solid-state asymmetric SC achieved an ED of 13.8 Wh kg^−1^. This nanohybrid can be considered a promising binder-free electrode for both aqueous and solid-state asymmetric SCs with these remarkable electrochemical properties.

## 1. Introduction

In recent times, mixed transition metal sulfides (MTMSs) have received tremendous attention for energy storage and conversion applications because of their unique properties, such as high electrochemical activities, rich redox reactions, and so forth. These MTMSs achieved higher electrical conductivity than their oxide counterparts due to a smaller bandgap [1,2,3]. Various MTMSs with prior stoichiometric ratios have been investigated as efficient candidates for multiple applications, including secondary batteries, supercapacitors (SCs), solar cells, electrocatalysis, fuel cells, and so on. [4,5,6,7,8,9]. Owing to their superior properties, such as high power density (PD), enhanced cycling stability, low maintenance cost, and environmentally friendly nature, SCs have been considered potential candidates to replace traditional energy resources (fossil fuels). Recently, the utilization of three-dimensional (3D) electrodes with porous nano-architectures such as carbon/graphene aerogel, carbon foam, 3D graphene networks, etc., has become a new direction of research as it provides efficient charge transfer and mass exchange during faradaic redox reactions by providing 3D interconnected networks. Using Ni foam as a 3D scaffold, a vast number of advanced SC electrodes have been constructed [10,11,12].

Several mixed transition metal oxides (MTMOs) in the last few years, including CuCo_2_O_4_, NiCo_2_O_4_, NiMn_2_O_4_, NiMoO_4_, CoMoO_4_, MnCo_2_O_4_, ZnCo_2_O_4_, etc., have been utilized as SC electrodes [13,14,15,16]. MTMSs have been introduced to improve the supercapacitive performance further. These MTMSs improved the electrical conductivity and enhanced the electrochemical performances. Among different MTMSs, NiCo_2_S_4_ has received remarkable attention as a promising electrode material due to its low cost, easy synthesis process, and high electrochemical stability [17,18,19,20]. Besides NiCo_2_S_4_, other ternary metal sulfides have also been explored as efficient SC electrodes. For example, Tang et al. reported a ternary FeNi_2_S_4_/CNT/graphene nanocomposite that exhibited a *C_s_* of 725 F g^−1^ at a current density of 10 A g^−1^. The electrode also showed capacity retention of 88% after 2500 charge-discharge cycles [21]. In another study, Kumar et al. demonstrated excellent electrochemical performance of NiV_2_S_4_ nanosheet arrays grown on Ni foam. The electrode displayed a specific capacity (*Q_s_*) of 639 C g^−1^ at 2 mA cm^−2^ and capacity retention of 90.7% at 30 mA cm^−2^ current density after 2000 cycles [22]. In a recent report, Fe-Co-S nanosheets were hydrothermally grown on graphene-coated Ni foam for developing SC electrodes. Benefited by the synergistic contribution from each component, this ternary nanohybrid displayed the *C_s_* of 850 F g^−1^ at 1 mA cm^−2^ current density [23]. In another work, Ni-Co sulfide nanotubes were combined with Ni-Co layered double hydroxide nanosheets on Ni foam through a synthetic hydrothermal route to develop a binder-free SC electrode [24]. Such electrodes exhibited the *C_s_* of 2105 F g^−1^ at the current density of 2 A g^−1^. Recently, Tung et al. developed an SC electrode by depositing rGO/CuCo_2_S_4_ nanocomposite (hydrothermally synthesized) on graphite paper through a 3D printing technique combined with a freeze gelation process [25]. This particular electrode type achieved the *C_s_* of 1123 F g^−1^ at the scan rate of 5 mV s^−1^ and cycling stability of 91% after 20,000 cycles. A recent report fabricated a solid-state symmetric SC device by combining MnCo_2_S_4_ with MnCo_2_O_4_ [26]. The device exhibited a good *Q_s_* of 417 C g^−1^ and cycling stability of 84.2% after 5000 cycles. On the other hand, Phonsuksawang et al. investigated the impact of Mn doping on NiCo_2_S_4_/Ni_3_S_2_ electrodes. The Mn-doped electrode exhibited a maximum *C_s_* of 1350 F g^−1^ [18].

Among MTMSs, although many reports are available on NiCo_2_S_4_ for various energy applications, less work has focused on MTMS composite materials consisting of molybdenum (Mo). It is expected that the exchange of oxygen of MTMOs with sulfur to form MTMSs can form flexible structures and enhance electrochemical performance by inhibiting the breakdown of the structure by elongation between layers during electrochemical testing [27]. In this aspect, Dai et al. synthesized CoMoS_4_ nanoparticles by a facile co-precipitation method. They obtained a *C_s_* of 415 F g^−1^ at a current density of 0.5 A g^−1^, displaying excellent capacitance retention of 100% after 10,000 cycles in 6M KOH electrolyte [28]. In another report, amorphous CoMoS_4_ exhibited a *C_s_* of 661 F g^−1^ at a current density of 1A g^−1^ in 1 M KOH electrolyte. A hybrid SC device based on this amorphous CoMoS_4_ and graphene exhibited excellent capacitance retention of 86% after 10,000 cycles [29].

Further, Yang et al. synthesized vertically aligned Co_3_S_4_/CoMo_2_S_4_ ultrathin nanosheets on reduced graphene oxide and investigated them for an SC electrode. The hybrid electrode exhibited a *C_s_* of 1457.8 F g^−1^ at the current density of 1A g^−1^ and excellent capacitance retention of 97% after 2000 cycles [30]. Recently, hierarchical Co-Mo-S nanosheets have been grown on Ni foam through a microwave-assisted hydrothermal process. Such mixed metal-sulfide-based binder-free electrodes displayed the *C_s_* of 1080 F g^−1^ at the current density of 1 A g^−1^ and 90.4% cycling stability after 5000 cycles [31]. In another work, hollow core-shell structured CoMoS_4_ was combined with Ni-Co-S nanotubes through the hydrothermal-assisted electrodeposition process. Such nano-arrays grown on carbon cloth displayed suitable specific capacitance of 2208.5 F g^−1^ at the current density of 1 A g^−1^ and cycling stability of 91.3% after 5000 cycles [32].

Inspired by these findings, we have grown CoMoS_4_ and Ni_3_S_2_ on Ni foam in a binderless electrode of SCs. Further, a conductive wrapping of MnO_2_ on CoMoS_4_/Ni_3_S_2_@Ni foam (NCMS) enhanced the electrochemical performance in an aqueous electrolyte. Most importantly, a strong synergy between the metal sulfide (Ni_3_S_2_), MTMS (CoMoS_4_), and metal oxide (MnO_2_) enhanced the capacitive properties of CoMoS_4_/Ni_3_S_2_/MnO_2_@Ni foam (NCMSM) heterostructures. Furthermore, aqueous and solid-state asymmetric SCs with superior electrochemical performance were constructed using this ternary composite. To the best of our knowledge, this is the first report on a CoMoS_4_-based ternary composite, which can be used for both aqueous and solid-state asymmetric SCs.

## 2. Experimental

### 2.1. Preparation of Materials

#### 2.1.1. Materials

Na_2_MoO_4_ dihydrate was purchased from Sigma Aldrich. Co(NO_3_)_2_ hexahydrate and thiourea were delivered by Alfa Aesar (USA). Sigma Aldrich (USA) supplied poly(vinyl alcohol). Other chemicals such as ethanol and KMnO_4_ were purchased from Duksan Pure Chemicals Co. Ltd. (Korea). All chemicals were used without further processing. Ni foam was purchased from MTI Corporation (USA).

#### 2.1.2. Preparation of Ni_3_S_2_/CoMoS_4_@Ni Foam (NCMS)

The CoMoS_4_-based electrode material was prepared by a one-step hydrothermal process followed by short annealing treatment. In a typical procedure, 2 mM of Co(NO_3_)_2_·6H_2_O and 2 mM of Na_2_MoO_4_·2H_2_O were dissolved in 40 mL of DI water by stirring. Then, 8 mM of thiourea was added to the solution, and the stirring was continued for another 1 h. After that, one piece of cleaned Ni foam (1 cm × 3 cm) was added to the solution, and the whole solution, along with the Ni foam, was transferred to a 50 mL Teflon-lined autoclave. Then, the autoclave was transferred to a heating oven and heated at 150 °C for 6 h. Before the hydrothermal process, the piece of Ni foam was cleaned with 6 M HCl and DI water to remove the NiO layer and other impurities from the surface. When the autoclave cooled down to room temperature, the precursor-coated Ni foam was washed with DI water and ethanol 2–3 times and dried at 60 °C for 6 h. Finally, the precursor-coated Ni foam was annealed at 300 °C in an Ar atmosphere for 1 h to obtain NCMS.

#### 2.1.3. Preparation of Ni_3_S_2_/CoMoS_4_/MnO_2_@Ni Foam (NCMSM)

For the preparation of NCMSM, the NCMS sample was placed in KMnO_4_ solution (2 mM of KMnO_4_ in 40 mL DI water). The solution was then transferred to a 50 mL autoclave and hydrothermally heated at 140 °C for 2 h. After washing and cleaning, the MnO_2_-coated Ni foam was annealed at 300 °C in the air for 1 h to obtain NCMSM nanohybrid.

#### 2.1.4. Preparation of Ni_3_S_2_@Ni Foam (NS)

For a comparative study, NS was prepared by a similar process to NCMS without adding Co and Mo precursors, i.e., Co(NO_3_)_2_·6H_2_O and Na_2_MoO_4_·2H_2_O.

#### 2.1.5. Asymmetric SC Devices Fabrication

NCMSM, reduced graphene oxide (rGO)-coated Ni foam (rGO@Ni foam), and PVA/KOH gel to fabricate the solid-state asymmetric SC device were used as the positive electrode, negative electrode, and electrolyte, respectively. The all-solid-state SC was prepared by assembling the negative and positive electrodes in a face-to-face alignment with the addition of nylon cloth as the separator. The aqueous asymmetric SC was accumulated with the same positive and negative electrodes separated by cellulose filter paper (with a thickness of 0.2 mm). The electrochemical test was performed in a 1 M KOH solution. Before testing the device, the charges of the positive and negative electrodes were balanced. The detailed synthesis process for the negative electrode is included in the supporting information.

#### 2.1.6. Characterization

X-ray diffraction (XRD) analysis of the electrode materials was performed using a PANalytical (Xpert-PRO MPD) instrument with a 2θ range of 10–80°. The surface morphology and microstructures of the test samples were observed by field emission scanning electron microscopy (FESEM, Hitachi (Japan), S-4800) and high-resolution transmission electron microscopy (HRTEM, Philips (The Netherlands), CM-200, at an acceleration voltage of 200 kV). X-ray photoelectron spectroscopy (XPS, Thermo Scientific, USA) was carried out using Al-Kα monochromatic radiation. The powders of electrode materials were detached from the Ni foam by sonication and collected. The surface area and pore size distribution measurements of these powders were performed in a Micromeritics 3Flex Surface Characterization Analyzer (Micromeritics Instrument Corp., USA) using the Brunauer–Emmett–Teller (BET) and Barrett–Joyner–Halenda (BJH) methods.

The electrochemical tests of the electrode materials were measured on an Autolab PGSTAT 302N instrument (Metrohm Autolab, The Netherlands) using a three-electrode cell with a working electrode, a platinum counter electrode, and a Ag/AgCl reference electrode. The electrode materials were used directly without adding any additive/binder at room temperature. KOH (1 M) aqueous solution was used as the electrolyte. The mass loadings of NS, NCMS, and NCMSM were 0.8, 1.5, and 2.1 mg cm^−2^, respectively. All the equations for calculating specific capacitance, energy density (ED), and PD are included in the electronic supplementary information.

## 3. Results and Discussion

### 3.1. Synthesis and Structural Analysis

The following equations explain the formation mechanism of individual components such as Ni_3_S_2_, CoMoS_4_, and MnO_2_ on Ni foam. Thiourea was decomposed in water during the hydrothermal process to form H_2_S, which reacted with sodium molybdate to form Na_2_MoS_4_. The Na_2_MoS_4_ further dissociated to form MoS_4_^2−^ ion, which is then associated with Co^2+^ to form CoMoS_4_. The formation of a red-colored solution after hydrothermal treatment indicated the formation of MoS_4_^2−^ ion [29,33]. The probable reactions are given below [34,35,36]:

(NH_2_)_2_CS + 2H_2_O → 2NH_3_ + CO_2_ + H_2_S (1)

4H_2_S + Na_2_MoO_4_ → Na_2_MoS_4_ + 4H_2_O (2)

Na_2_MoS_4_ → 2Na^+^ + MoS_4_^2^^−^ (3)

Co^2+^ + MoS_4_^2^^−^ → CoMoS_4_ (4)

During the decomposition of thiourea, H_2_S formed, which further reacted with the outer surface of Ni foam to form Ni_3_S_2_ on the Ni foam [36].

2H_2_S + 3Ni → Ni_3_S_2_ + 2H_2_ (5)

The formation of MnO_2_ was based on the following reaction.

4MnO_4_^−^ + 2H_2_O → 4MnO_2_ + 4OH^−^ + 3O_2_ (6)

**Figure 1:**
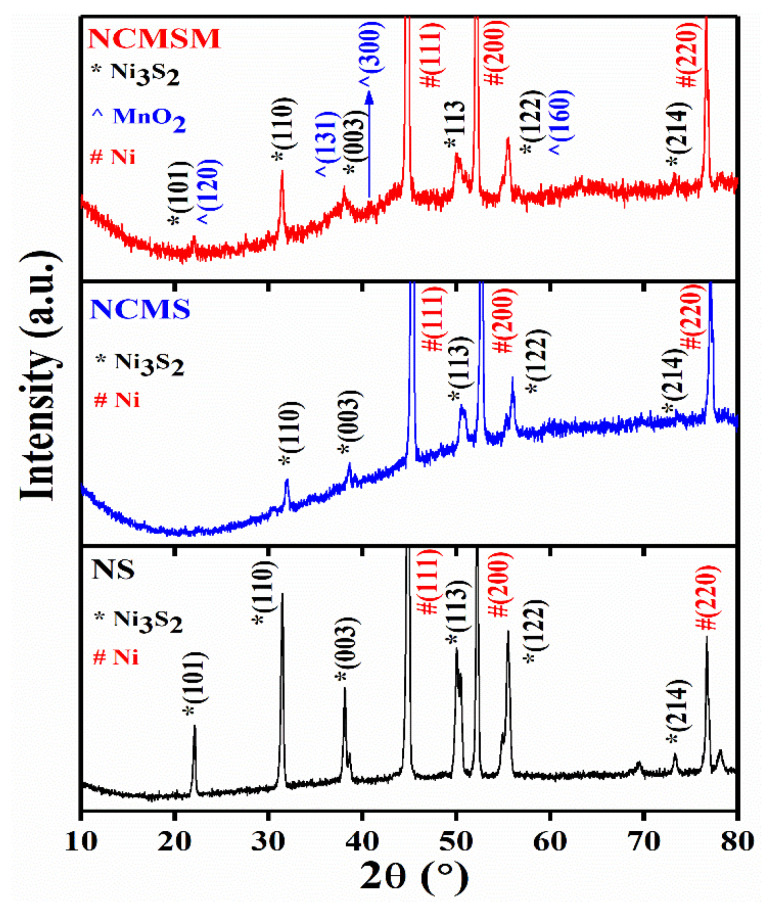
XRD patterns of NS, NCMS, and NCMSM samples.

To confirm the formation of individual components, XRD analysis was performed on NS, NCMS, and NCMSM. As observed in Figure 1, NS showed characteristic peaks of Ni_3_S_2_ along with the three major peaks of Ni foam. Peaks at 2θ = 22.1, 31.3, 38.1, 50.0, 55.5, and 73.3° could be indexed to the (101), (110), (003), (113), (122), and (214) planes of Ni_3_S_2_, respectively (JCPDS Card No. 73-0698) [34]. The XRD pattern of NCMS showed only characteristic peaks of Ni_3_S_2_. It is important to note that no peak was observed for CoMoS_4_ due to its amorphous nature. This result is consistent with previous results [32,33,34]. However, the XRD pattern of NCMSM nanohybrid showed characteristic peaks of Ni_3_S_2_ and MnO_2_. The characteristic peaks at 2θ = 22.1, 38.6, 40.8, and 55.5° nearly matched the (120), (131), (300), and (160) planes of γ-MnO_2_ according to JCPDS Card No. (14-0644). Most importantly, γ-MnO_2_ showed low-intensity peaks, most of which overlapped with the characteristic peaks of Ni_3_S_2_. The low-intensity peaks resulted from the poor crystallinity of γ-MnO_2_. This result is supported by previous literature [35]. Therefore, XRD analysis confirmed the presence of MnO_2_, CoMoS_4_, and Ni_3_S_2_ in NCMSM.

**Figure 2:**
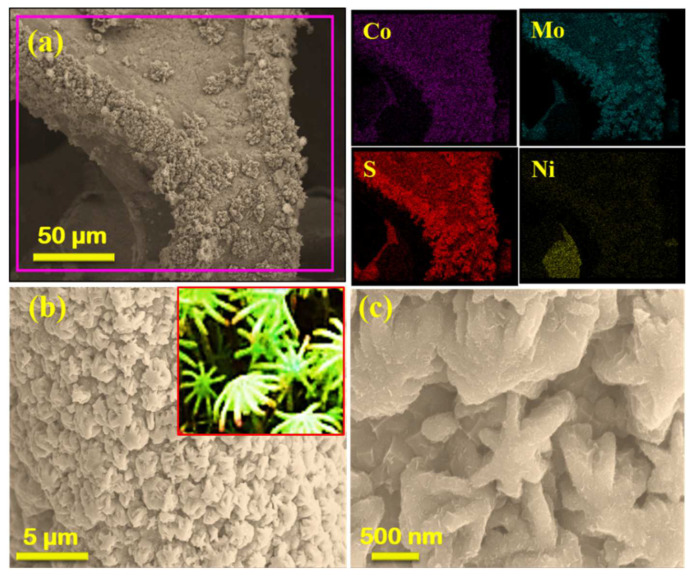
SEM images of NCMS at different magnifications (**a**–**c**) and the elemental mapping of (**a**) for Co, Mo, S, and Ni. The inset in (**b**) is a picture of female sporocarps, similar to the morphology of NCMS in (**c**).

**Figure 3:**
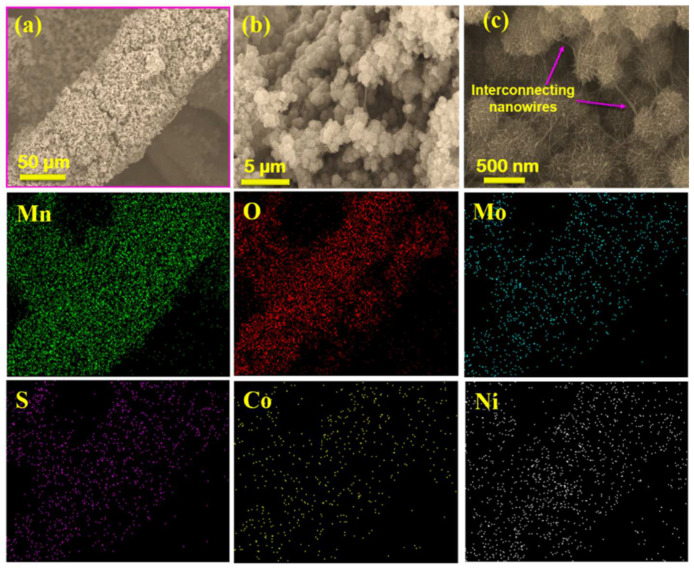
SEM images of NCMSM at different magnifications (**a**–**c**) and the corresponding elemental mapping for Mn, O, S, Mo, Co, and Ni.

The morphologies of the as-prepared electrode materials were investigated by FESEM. Figure 2a–c shows that the Ni foam was covered with polyhedral CoMoS_4_ in NCMS. The morphology is similar to the female sporocarps of the common liverwort plant, as shown in the inset of Figure 2b. Further, the elemental mapping confirms a uniform distribution of Co, Mo, S, and Ni in NCMS. Figure 3a–c shows the FESEM images of NCMSM heterostructures, which indicate the wrapping of porous MnO_2_ nanowires on NCMS. It has been observed that the nanoflakes of MnO_2_ entirely covered the surface of the electrode material with interconnecting nanowires of an average diameter of 10–20 nm (Figure 3b). This type of interconnected nanowire can facilitate easy and efficient electrolyte transport to the interior of the electrodes by creating ample open spaces and sufficient electroactive sites [36].

In addition, this type of mesoporous structure increases the electrolyte-accessible surface area and enhances the charge-storage ability. The corresponding elemental mapping of NCMSM is also shown in Figure 3, which indicates the presence of Mn, O, Mo, Co, S, and Ni. For comparison, the surface morphology of NS was also investigated, and the corresponding SEM images are shown in Figure S1c,d. Figure S1a,b shows the low- and high-magnification SEM images of the bare Ni foam, which indicate the smooth surface of the foam. This smooth surface became rough in NS with the formation of porous granules of Ni_3_S_2_. The mechanism of creating this unique morphology can be explained by the nanoscale Kirkendall effect, reported previously [37].

**Figure 4:**
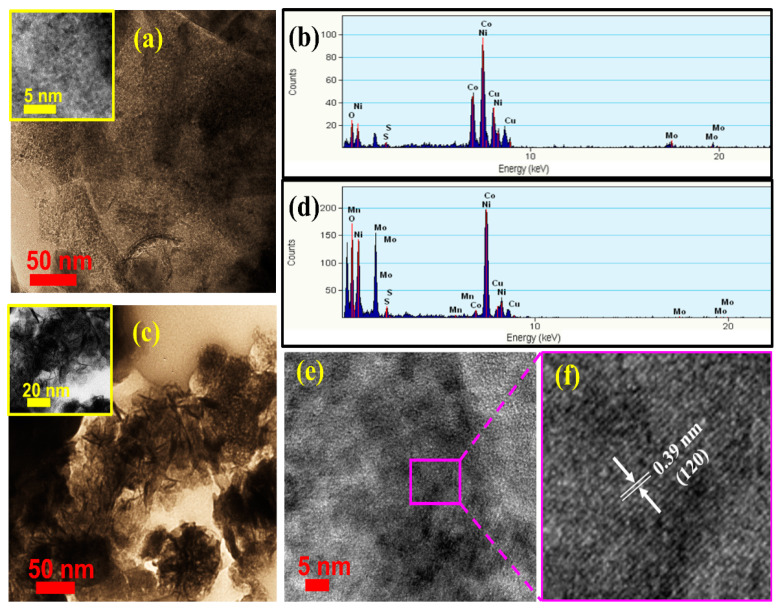
(**a**) TEM image (inset—high-magnification image) and (**b**) the corresponding EDX spectrum of NCMS; (**c**) TEM image (inset: high-magnification image) and (**d**) the corresponding EDX spectrum of NCMSM; (**e**,**f**) HRTEM image of NCMSM showing the lattice fringes of (120) planes.

To investigate the microstructures of NCMS and NCMSM by HRTEM, the coated Ni foams were ultrasonically dispersed in ethanol and then deposited on a Cu grid. The TEM image of NCMS shows a sheet-like morphology, which might be formed by breaking down the polyhedral structure of CoMoS_4_ during sonication (Figure 4a). On the other hand, in the high-magnification image of NCMS, no prominent diffraction fringes are observed, which can be attributed to the amorphous nature of CoMoS_4_ (inset, Figure 4a). A similar observation was also reported for CoMoS_4_ [29]. The corresponding EDX spectrum of NCMS is shown in Figure 4b, which indicates the presence of Co, Mo, S, and Ni. A TEM image of NCMSM heterostructures revealed the agglomeration of MnO_2_ nanoflakes (Figure 4c). These ultrathin nanoflakes are agglomerated to form silky porous channels (Figure S2b). The thicknesses of the individual nanoflakes are ranged from 2 to 3 nm. Similar morphology was also reported in a previous MnO_2_-based article [38]. This type of porous nanostructure can shorten the ion diffusion pathway and enhance electrochemical performance. On the other hand, the ring-like SAED pattern indicates the polycrystalline nature of MnO_2_ (Figure S2a). Further, the EDX spectrum confirms the presence of the individual elements (Figure 4d). It is important to note that the HRTEM image shows the lattice fringes of ~0.39 nm, corresponding to the (120) plane of γ-MnO_2_ (Figure 4e,f). Therefore, HRTEM results are in good agreement with the XRD data.

**Figure 5:**
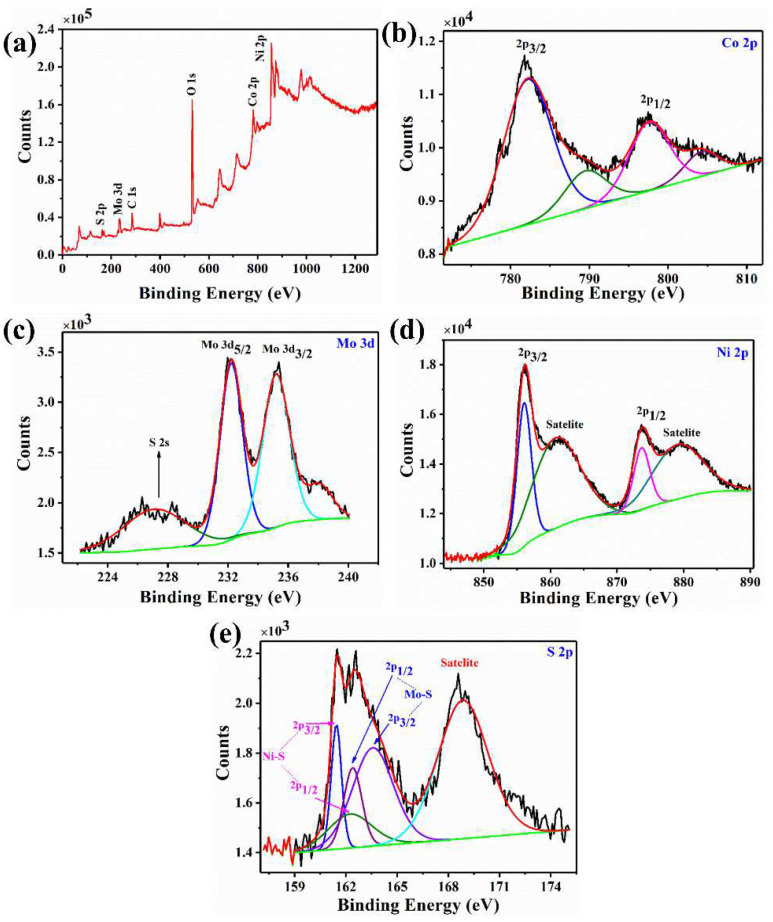
XPS of NCMS: (**a**) survey spectrum and (**b**) Co 2p, (**c**) Mo 3d, (**d**) Ni 2p, and (**e**) S 2p core-level spectrum.

The chemical composition of NCMS and NCMSM was investigated by XPS analysis. The survey spectrum indicated the presence of Ni, Co, Mo, and S in NCMS (Figure 5a). Additionally, C 1s (as reference) and O 1s peaks were due to air exposure. The Co 2p core-level spectrum presented two significant peaks at binding energies 782.3 and 797.6 eV, corresponding to 2p_3/2_ and 2p_1/2_ spin-orbit peaks (Figure 5b). Furthermore, the Mo 3d high-resolution spectrum showed a shoulder peak at the binding energy of 227.4 eV, which corresponded to S 2s photoelectrons (Figure 5c) [39,40]. Besides this, a doublet of peaks appeared at 232.2 and 235.2 eV, which can be assigned to Mo 3d_5/2_ and Mo 3d_3/2_, respectively [29,41]. Significantly, the binding energies of 3d shifted slightly, suggesting the interaction between Ni_3_S_2_ and CoMoS_4_. In addition, the presence of Mo 3d_5/2_ and Mo 3d_3/2_ peaks confirms the VI oxidation state of Mo [28]. In the Ni 2p spectrum, two spin-orbit doublets and two shake-up satellites were observed (Figure 5d). The peaks at binding energies of 856.2 and 874.0 eV could be attributed to Ni 2p_3/2_ and Ni 2p_1/2_, respectively. Figure 5e shows the high-resolution S 2p spectrum, which was deconvoluted into four prominent peaks and one shake-up satellite peak at 168.8 eV. The peaks at 163.5 and 162.3 eV were assigned to S 2p_3/2_ and S 2p_1/2_ of the Mo-S bond, respectively [42]. Furthermore, the peaks at 162.2 and 161.4 eV were attributed to S 2p_3/2_ and S 2p_1/2_ of the Ni-S bond. Therefore, XPS analysis of NCMS confirmed the successful synthesis of CoMoS_4_ and Ni_3_S_2_.

**Figure 6:**
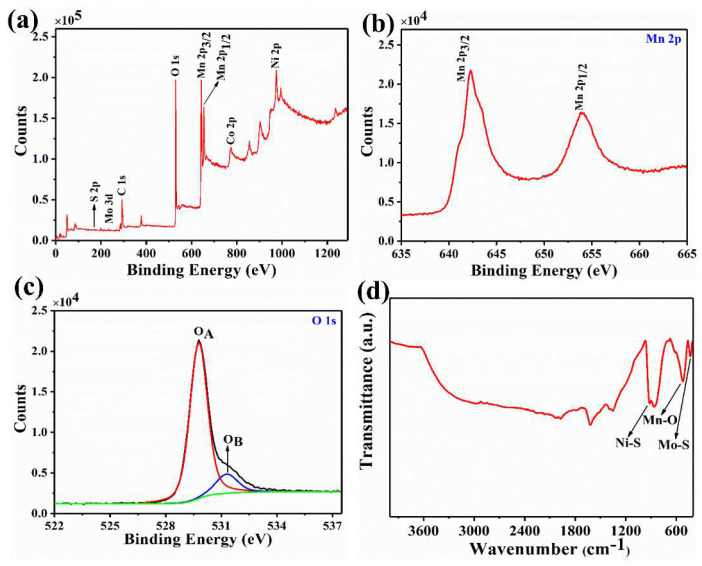
XPS survey spectrum of NCMSM: (**a**) survey spectrum; (**b**) Mn 2p; and (**c**) O 1s core-level spectra; (**d**) FTIR spectrum of NCMSM.

Figure 6a–c show the XPS spectra of NCMSM heterostructures. The survey spectrum showed characteristic peaks of individual elements such as S, Mo, C, O, Mn, Co, and Ni (Figure 6a). Due to proper wrapping of MnO_2_, low-intensity peaks were observed from S 2p, Mo 3d, Co 2p, and Ni 2p. The core-level spectrum of Mn 2p showed Mn 2p_3/2_ and Mn 2p_1/2_ peaks with binding energies of 642.3 and 653.9 eV. However, the spin-energy separation between these two peaks was 11.6 eV, which agrees with previous reports [43,44]. The deconvolution of the O 1s spectrum showed two prominent peaks. The peak at binding energy of 528–530 eV was attributed to surface lattice oxygen (O_A_), and the other at binding energy of 531–532 eV was assigned to surface chemisorbed oxygen (O_B_) [45,46]. The XPS spectrum of NS also confirmed the presence of Ni 2p and S 2p levels (Figure S3a). Further, the FTIR spectrum was recorded to check the chemical environment of NCMSM (Figure 6d). The peak at wavenumber < 500 cm^−1^ corresponds to the vibrational mode of the Mo-S bond [47]. A sharp peak at 520.3 cm^−1^ can be attributed to the characteristic Mn-O bond of MnO_2_ [48]. On the other hand, another pronounced peak at 914.6 cm^−1^ represents a distinct Ni-S bond, indicating the presence of Ni_3_S_2_ [49]. In addition, other small peaks centered at 689, 862, and 1621 cm^−1^ correspond to C-H bending, N-H bending, and O-H stretching vibration modes, respectively. The appearance of these peaks indicates the presence of impurities (from the precursor salts) and moisture. Lastly, the small peak at 1390 cm^−1^ appeared due to the formation of metal sulfates. Therefore, the FTIR spectrum confirms the presence of two different kinds of metal-sulfur (Mo-S and Ni-S) and metal-oxygen (Mn-O) bonds. Therefore, the XPS and FTIR analysis confirmed the presence of MnO_2_ along with other components such as CoMoS_4_ and Ni_3_S_2_ in NCMSM.

N_2_ adsorption-desorption analysis was performed with the electrode powder (detached from the Ni foam) to evaluate the porous nature. The N_2_ adsorption-desorption isotherm and the pore-size distribution curves for NCMS and NCMSM are shown in Figure S4a–d. Both electrodes showed a typical type-IV isotherm profile with a hysteresis loop, indicating mesoporous features. NCMSM heterostructures exhibited higher nitrogen uptake capacity than NCMS, which means higher porosity. The BET surface area of NCMS was measured to be 12.22 m^2^ g^−1^, which is increased to 32.77 m^2^ g^−1^ for NCMSM. The increased surface area of NCMSM is attributed to the porous coating of MnO_2_ interconnected nanowires on NCMS. The average pore size of NCMS was 24.45 nm, while NCMSM showed an average pore size of 8 nm. The pore-size distribution curves show that a few macropores were also present in both electrode materials along with the mesopores. Most importantly, both these mesopores and macropores can significantly enhance the transport of electrolyte ions to the electrodes and improve the diffusion of electrons by providing an easy transport pathway [50].

**Figure 7:**
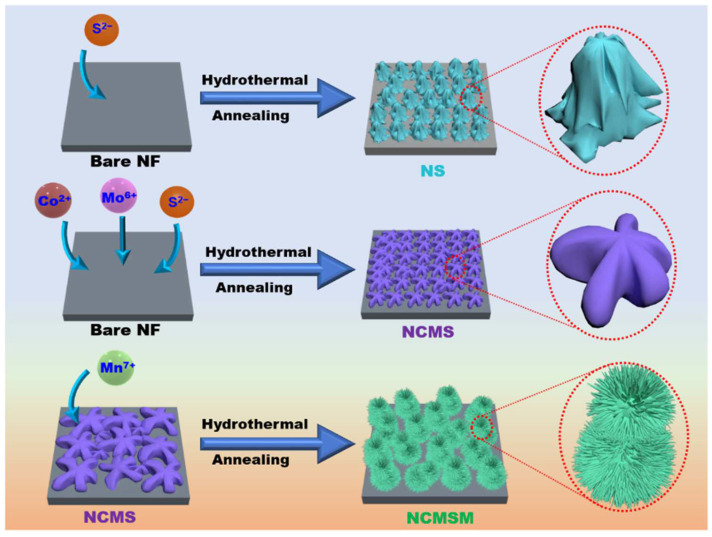
Schematic diagram showing the formation of NS, NCMS, and NCMSM.

Figure 7 presents a schematic illustration of the growth mechanism of NS, NCMS, and NCMSM based on structural and morphological analyses. For NS, during the hydrothermal reaction, the elemental Ni of Ni foam reacted with thiourea to form Ni_3_S_2_ primary particles (Equation (5)). In addition, these primary particles aggregated randomly, self-assembled, and formed porous granules to reduce their surface area. On the other hand, for NCMS, the precursor ions were mixed and adsorbed on the Ni foam. Under hydrothermal conditions, the metallic Ni reacted with sulfur to form Ni_3_S_2_ according to the exact growth mechanism for NS. Then, the surface of the Ni_3_S_2_ that was coated on Ni foam was entirely covered with the CoMoS_4_ precursors. In addition, the metal ions reacted with S^2−^ to form CMS primary particles. Finally, the primary particles self-assembled and developed a common liverwort plant-like morphology through an anisotropic growth process. In the case of NCMSM nanohybrid, the MnO_2_ precursor nanoparticles were first formed and adsorbed on the Ni foam. As the reaction proceeded, the growth process transformed into a kinetically controlled process [51]. Under these conditions, the nanoparticles began to self-assemble, forming nanoflakes. Moreover, as the reaction proceeded, some nanoflakes continued to grow and converted to nanowires and interconnected with each other. At the end of the reaction, the entire Ni foam surface was fully coated with this porous nano-architecture, as shown in the schematic diagram. This type of porous nano-architecture is highly desirable for superior electrochemical performance because of the large surface area.

### 3.2. Electrochemical Characterization

**Figure 8:**
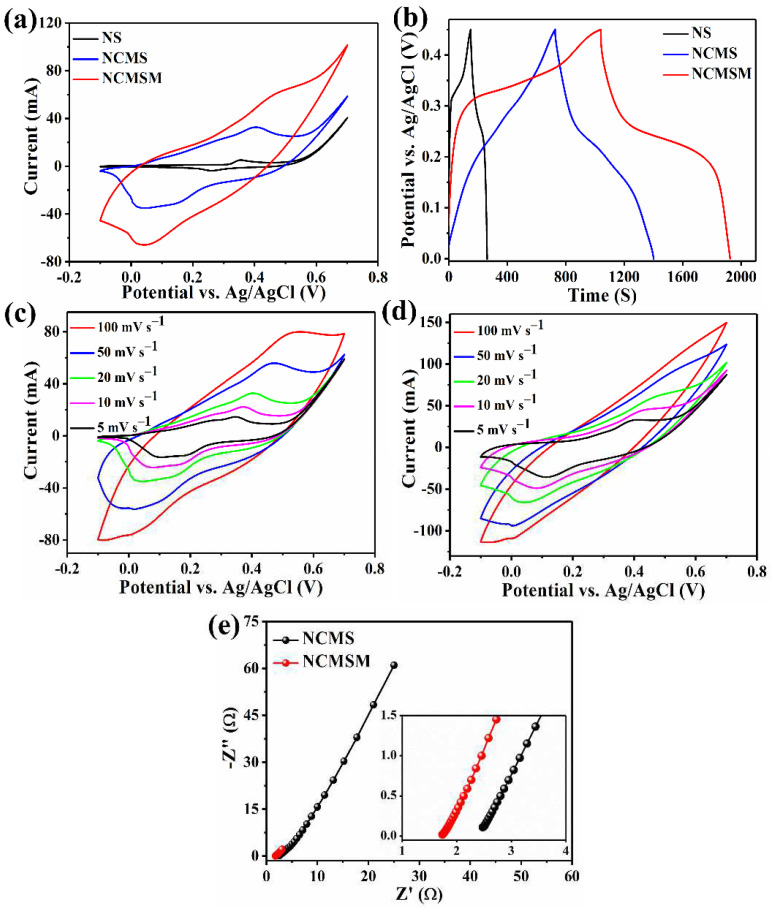
(**a**) CV curves at a scan rate of 20 mV s^−1^ within the potential window −0.1 to 0.7 V and (**b**) galvanostatic charge-discharge profiles at a current density of 1 A g^−1^ within the potential window 0 to 0.45 V for NS, NCMS, and NCMSM; CV curves of (**c**) NCMS and (**d**) NCMSM at different scan rates within the potential window −0.1 to 0.7 V; and (**e**) Nyquist plots of NCMS and NCMSM (inset: a magnified view of the high-frequency region); all measured in 1 M KOH.

The electrochemical performances of the electrode materials were investigated in 1 M KOH. Figure 8a compares the CV curves of NS, NCMS, and NCMSM electrodes, measured at the scan rate of 20 mV s^−1^ in the potential window from −0.1 to 0.7 V. All the electrode materials exhibited a pair of redox peaks due to their faradaic capacitive behavior. Importantly, NCMSM showed a higher current response than the other electrodes, indicating superior electrochemical performance. A comparison of charge-discharge profiles of these electrodes at the current density of 1A g^−1^ is presented in Figure 8b. NCMSM heterostructures displayed a much longer charging-discharging time than others, suggesting their preferable electrochemical properties. As calculated from the discharge profile, NCMSM heterostructures exhibited a high *C_s_* of 2021 F g^−1^ (*Q_s_* of 909.5 C g^−1^, which corresponds to ~245.5 mAh g^−1^) at the current density of 1 A g^−1^ in the potential window from 0 to 0.45 V. These values are higher than CMSM (*C_s_* of 1333 F g^−1^ and *Q_s_* of 600 C g^−1^, which correspond to 162 mAh g^−1^) and NS (*C_s_* of 295 F g^−1^ and *Q_s_* of 133 C g^−1^, which correspond to ~36 mAh g^−1^). From the capacitance values, it is observable that Ni_3_S_2_ contributed little to the charge-storage process. However, CoMoS_4_ exhibited superior capacitive properties. The conducting wrapping of MnO_2_ interconnected nanowires made a noticeable contribution to the electrochemical performance. The *C_s_* of the NCMSM nanohybrid is higher than the number of MnO_2_-based electrode materials reported previously (Table S1). The following reasons can explain the enhanced capacitive properties of NCMSM: (1) the interconnected nanowire network structure of MnO_2_ might have provided efficient pathways for smooth and effective electron transport; (2) strong synergy between the individual components such as CoMoS_4_ and MnO_2_; (3) easy and fast electrolyte ion (OH^−^) transport through the porous nano-architectures of the electrode material; (4) the growth of MnO_2_ nanowires on the conductive CoMoS_4_ on Ni foam can reduce the charge transfer barrier and enhance the mechanical adhesion between these materials [52].

The CV curves at different scan rates ranging from 5 to 100 mV s^−1^ for NCMS and NCMSM are shown in Figure 8c,d. No significant change was observed in the CV curves with the increase in the scan rate for both the electrodes, indicating ideal capacitive behavior. It is important to note that while the anodic peak shifted towards a higher positive potential, the cathodic peak moved to the high negative potential region with an increasing scan rate. This phenomenon can be ascribed to the polarization initiated by the increase in scan rate. At the low scan rate of 5 mV s^−1^, both NCMS and NCMSM exhibited a redox couple within the potential range of 0.05–0.4 V, resulting from the redox reactions associated with M-S/M-S-OH (M = Ni, Co). The faradaic reactions can be expressed as follows [30,53]:

Ni_3_S_2_ + 3OH^−^ → Ni_3_S_2_(OH)_3_ + 3e^−^ (7)

CoS + OH^−^ → CoSOH + e^−^ (8)

The Mo atoms do not participate in any redox reaction [30]. The CV curves of NS at different scan rates are shown in Figure S3b. NS exhibited a lower current response than NCMS and NCMSM due to the poor electrochemical activity of Ni_3_S_2_.

EIS analysis investigated the electrochemical activity further, and the Nyquist plots of NCMS and NCMSM are displayed in Figure 8e. Both impedance spectra show a straight line in the low-frequency region and a depressed semicircle in the high-frequency area. The *X*-axis intercept indicates the equivalent series resistance (ESR), which is generally associated with the resistance of electrode materials. NCMSM heterostructures exhibited a lower ESR value (1.73 Ω) than NCMS (2.47 Ω). This low value of ESR can be explained by the effective conductive coating of MnO_2_, which reduces the internal resistance of the electrode and interconnected nanowire morphology of MnO_2_, reducing the contact resistance between the electrode and electrolyte. The straight line in the low-frequency region indicates the Warburg impedance, which is generally related to the diffusion of electrolyte ions in the electrode materials. NCMSM showed lower Warburg impedance than NCMS because of its mesoporous structure, enabling easy diffusion of electrolyte ions to the electrode’s pores [54]. It is important to note that the straight line in the low-frequency region of the Nyquist plot of NCMS is more nearly vertical and parallel to the imaginary axis, indicating a high charge-discharge rate [55].

**Figure 9:**
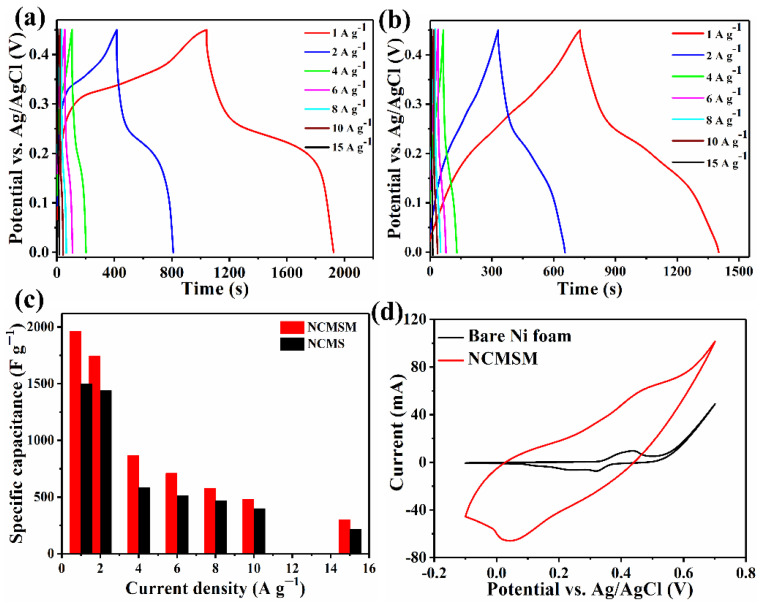
Galvanostatic charge-discharge curves of (**a**) NCMSM and (**b**) NCMS at different current densities within the potential window 0 to 0.45 V; (**c**) the histogram of *C_s_* vs. current density for NCMS and NCMSM; and (**d**) the CV curves of NCMSM and bare Ni foam at a scan rate of 20 mV s^−1^; all in 1M KOH.

Figure 9a,b represent the charge-discharge profiles of NCMSM and NCMS in the potential range 0–0.45 V. The nonlinear charge-discharge profiles indicate the existence of faradaic reactions during the charge-discharge process. A comparison of *C_s_* at different current densities for NCMS and NCMSM is shown in Figure 9c. NCMSM heterostructures exhibited specific capacitances of 2021, 1626, 714, 515.5, 371, 273, and 164.5 F g^−1^ at the current densities of 1, 2, 4, 6, 8, 10, and 15 A g^−1^, respectively. These results are higher than NCMS (1333, 1238.5, 395, 304, 234.5, 168, and 114 F g^−1^) at the same discharge current densities. With increasing current density, the specific capacitance decreased due to the enhancement of the voltage drop and deficiency of the active material to take part in the redox reaction [53]. Figure 9d shows the CV curves of NCMSM and bare Ni foam at the scan rate of 20 mV s^−1^. From the CV curves, we can notice that the contribution of bare Ni foam to the capacitance/capacity of the target electrode is negligible.

**Figure 10:**
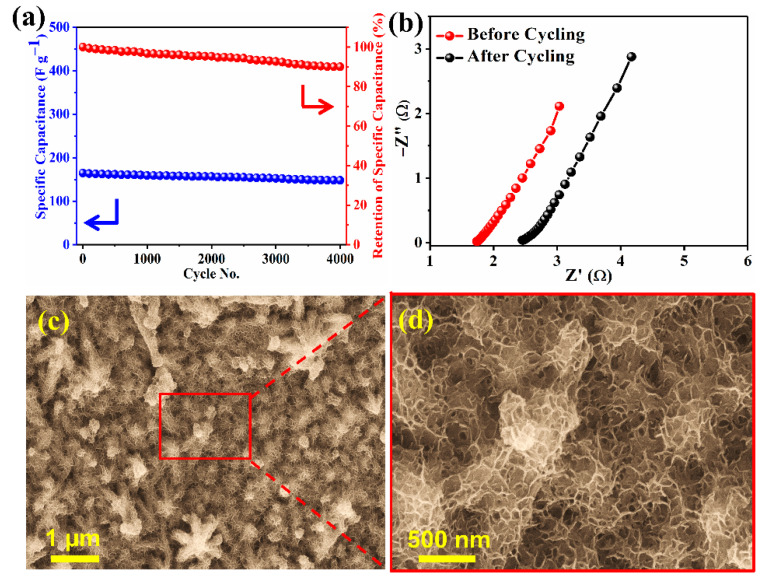
(**a**) Galvanostatic charge-discharge cycling performance at the current density of 15 A g^−1^ and (**b**) Nyquist plot before and after cycling; (**c**,**d**) SEM images at different magnifications, taken after 4000 cycles; all for NCMSM in 1 M KOH.

The cycling stability test for NCMSM heterostructures was performed over 4000 charge-discharge cycles at a constant current density of 15 A g^−1^, and the results are shown in Figure 10a. After 4000 consecutive cycles, 90% of the initial capacitance was retained. Decay in capacitance is associated with the structural deformation and desolation of active materials during cycling. The surface morphology of the active electrode material after charge-discharge cycles is shown in Figure 10c,d, which indicates the termination of MnO_2_ nanowires from the electrode surface. This high retention of capacitance can be explained by the decoration of MnO_2_ nanowires on the electrode surface, which increased the surface-to-volume ratio of the electrode and provided open channels for the easy ion adsorption and fast intercalation/deintercalation of electrolyte ions. The electrode material exhibited better cycling stability than several MnO_2_-based electrodes (Table S2). Figure 10b presents the Nyquist plots of NCMSM before and after the cycle test. The enhancement of the ESR value (2.477 Ω) after cycling can be attributed to the partial removal of conductive MnO_2_. Therefore, NCMSM heterostructure can be considered a good candidate for SC applications with this high cycling stability. To check the electrochemical activity of CoMoS_4_ without the interference of Ni_3_S_2_, CoMoS_4_ was grown on a carbon cloth substrate following a similar synthetic procedure, and the results are thoroughly discussed in the supporting information section (Section S3 and Figures S5 and S6).

**Figure 11:**
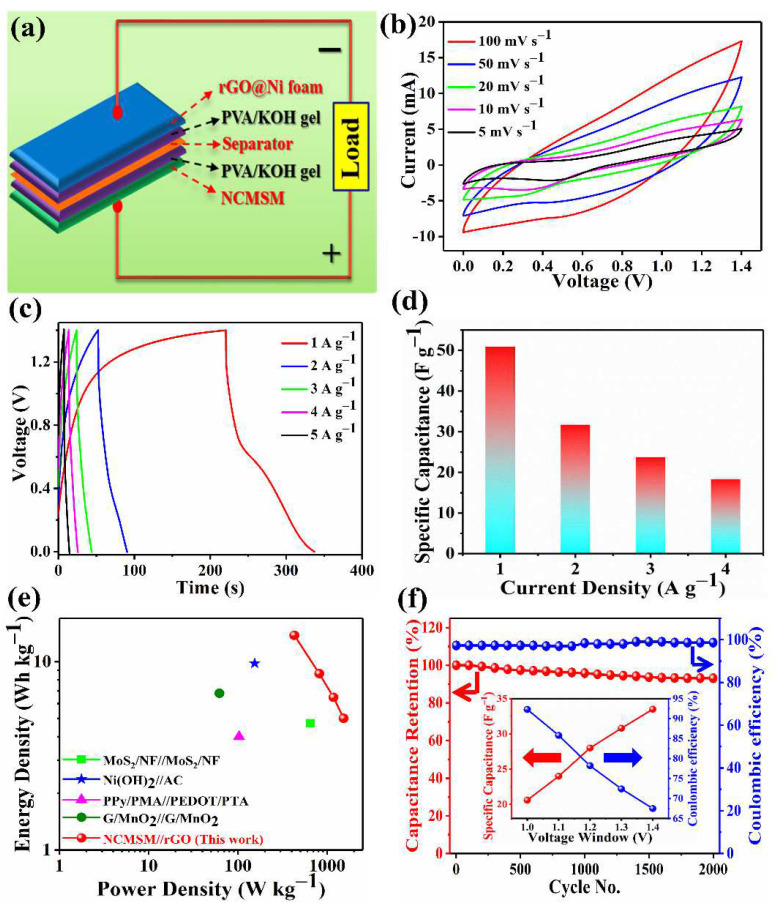
Electrochemical performance of a solid-state asymmetric SC (SASC, NCMSM//rGO): (**a**) schematic diagram of the device, (**b**) CV curves at various scan rates within a fixed potential window of 1.4 V, (**c**) galvanostatic charge-discharge curves at several current densities, (**d**) histogram of *C_s_* as a function of current density, (**e**) Ragone plot, and (**f**) cycling stability curve (measured at the current density of 5 A g^−1^); the inset shows the specific capacitance of the device at different potential windows from 1 to 1.4 V.

To examine the practical applicability of NCMSM, both aqueous and solid-state asymmetric SCs were fabricated with NCMSM and rGO@Ni foam as the positive and negative electrodes, respectively. The high- and low-magnification SEM images of rGO@Ni foam confirmed the coverage of graphene sheets over Ni foam (Figure S7). Figure 11a presents a solid-state asymmetric SC’s (SASC) schematic diagram. Figure S8a presents the CV curves at different potential windows at a constant scan rate of 100 mV s^−1^. The ASC exhibited almost rectangular CV curves, indicating capacitive behavior, even for high potential windows. On the other hand, at a potential window of 1.6 V, a large deviation from the rectangular shape was observed. Therefore, we chose a stable potential window of 1.4 V to evaluate the device’s electrochemical performance, which was then confirmed by the charge-discharge curves within the potential range of 0 to 1.4 V (Figure S8b). The CV curves of SASC at different scan rates within a stable potential window of 1.4 V are shown in Figure 11b. The quasi-rectangular CV curves indicate that the SASC can effectively operate at an operating voltage of 1.4 V. The nonlinear charge-discharge profiles indicate a faradaic reaction during the electrochemical test (Figure 11c). The corresponding *C_s_* values at different current densities are shown in Figure 11d. The device delivered a moderate *C_s_* of 51 F g^−1^ (*Q_s_* of 25.1 mAh g^−1^) at the current density of 1 A g^−1^, even higher than a series of ASCs (Table S3). Even at a high current density of 5 A g^−1^, the device still obtained the *C_s_* value of 10.6 F g^−1^. Figure 11e presents the Ragone plot of the SASC within the potential window of 1.4 V. The device delivered an ED of 13.8 Wh kg^−1^ at the PD of 429.3 W kg^−1^. Importantly, the SASC achieved a higher ED than a series of SC devices, such as MoS_2_/Ni foam//MoS_2_/Ni foam (4.7 Wh kg^−1^) [56], β-Ni(OH)_2_//activated carbon (AC) (9.8 Wh kg^−1^) [57], polypyrrole/phosphomolybdic acid//poly(3,4-ethylene dioxythiophene)/phosphotungstic acid (PPy/PMA//PEDOT/PTA) (4 Wh kg^−1^) [58], and graphene (G)/MnO_2_ //graphene/MnO_2_ (6.8 Wh kg^−1^) [59]. Cycling stability is one of the key parameters for evaluating the electrochemical performance of asymmetric SCs. The cycling stability of SASC was tested using a continuous charge-discharge test for 2000 cycles at a current density of 5 A g^−1^. After 2000 cycles, the SASC retained ~93% of its initial capacitance with a coulombic efficiency of 98.5% (Figure 11f). The inset in Figure 11f shows the *C_s_* and coulombic efficiency of the device at different potential windows from 1 to 1.4 V. The *C_s_* was increased, but the coulombic efficiency decreased with increasing potential window. The enhanced capacitance was attributed to increased charge accumulation with increasing potential. On the other hand, the decrease in coulombic efficiency was attributed to the evolution of H_2_ [29].

**Figure 12:**
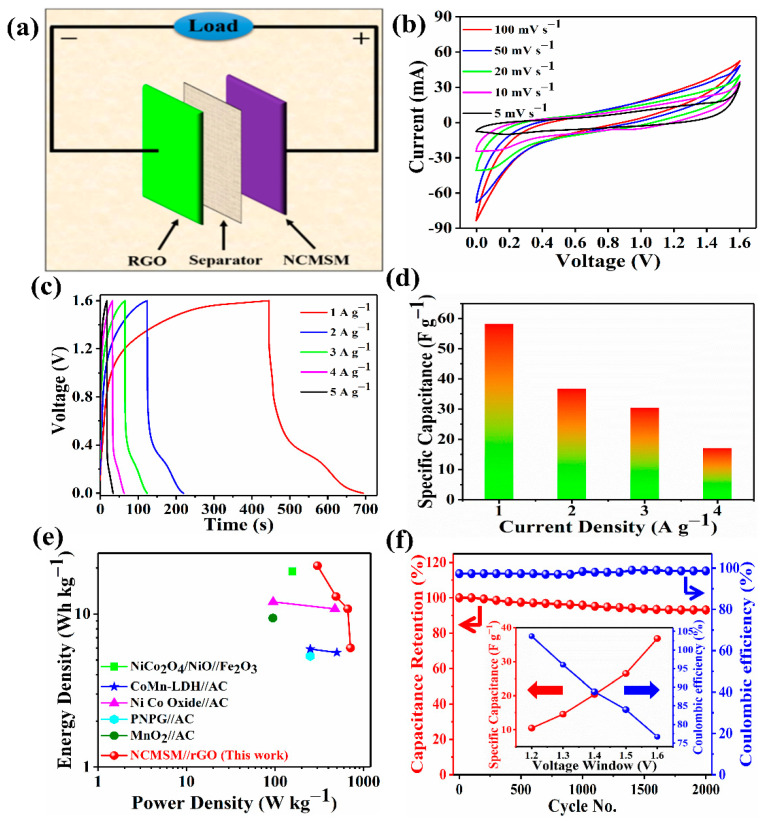
Electrochemical performance of the as-prepared aqueous asymmetric SC (AASC, NCMSM//rGO): (**a**) schematic diagram of the device, (**b**) CV curves at various scan rates within a fixed potential window of 1.6 V, (**c**) galvanostatic charge-discharge curves at several current densities, (**d**) histogram of the *C_s_* as a function of the current density, (**e**) Ragone plot, and (**f**) cycling stability curve (measured at the current density of 5 A g^−1^); the inset shows the *C_s_* of the device at different potential windows from 1.2 to 1.6 V.

The potential of NCMSM heterostructures was extended to construct an aqueous asymmetric SC (AASC). Figure 12a presents a schematic diagram of the device. The CV curves at different scan rates showed a slight deviation from a regular rectangular shape, which may be caused by the rich redox reaction (Figure 12b). An extended potential window of 1.6 V was chosen for the aqueous SC device based on the CV curves at 100 mV s^−1^ within different potential windows (Figure S8c), which was justified further by the identical charge-discharge profiles at different potential windows, as shown in Figure S8d. The charge-discharge profiles at various current densities also showed a similar trend to the solid-state device (Figure 12c). As expected, the device’s capacitance was decreased with increasing current density (Figure 12d). The AASC exhibited a high *C_s_* of 58.3 F g^−1^ (*Q_s_* of 25.1 mAh g^−1^) at a current density of 1A g^−1^, higher than the SASC. Furthermore, the value was higher than several mixed metal oxide/sulfide-based aqueous asymmetric SCs (Table S4). Figure 12e presents a Ragone plot of the AASC. The device exhibited a high ED of 20.7 Wh kg^−1^ at a PD of 301.4 W kg^−1^. The AASC showed a higher ED than the few reported aqueous asymmetric SCs, including NiCo_2_O_4_/NiO//Fe_2_O_3_ (19 Wh kg^−1^) [60], CoMn-LDH//AC (5.9 Wh kg^−1^) [61], NiCo oxide//AC (12 Wh kg^−1^) [62], poly(N-phenyl glycine)//AC (5.3 Wh kg^−1^) [63], and MnO_2_//AC (9.4 Wh kg^−1^) [64]. Furthermore, the AASC displayed remarkable cycling stability of ~96% with excellent coulombic efficiency of ~99% after 2000 charge-discharge cycles (Figure 12f). Similar to the solid-state device, the specific capacitance increased, and the coulombic efficiency decreased with increasing current density for AASC, as shown in the inset of Figure 12f.

A detailed comparison of the electrochemical performance of these two types of devices is shown in Table 1. The overall electrochemical performance of the aqueous device was slightly higher than the solid-state device, which could be attributed to the following: (1) the increase in operating potential resulted in a higher ED for the AASC as the ED is directly proportional to the square of the operating potential. Moreover, the capacitance of the AASC was enhanced due to the low internal resistance, fast electron transport, and high charge accumulation in the presence of an aqueous electrolyte. (2) The aqueous electrolyte enabled smoother and more rapid electron and ion transport than the solid-state electrolyte. (3) The contact between the electrode and electrolyte was smoother for the aqueous device, enhancing conductivity. For the SASC, a decrease in contact resistance between the electrode and electrolyte during the cycling test resulted in capacity fading.

Table 1 differentiates the electrochemical performance of two kinds of asymmetric devices. The device with an aqueous electrolyte exhibited high electrochemical properties. However, the solid-state one still showed moderate performance in addition to its higher temperature use and prevention of short circuits, which makes it commercially viable. We believe that this kind of comparative study is necessary to explore the potential commercialization of the SC electrode materials. Lastly, these smart combinations of metal sulfides and oxides can also be explored for other energy-related applications, including Li-ion batteries, fuel cells, etc.

Moreover, to identify the contribution of bare NF to the electrochemical performance of the electrode materials, the capacity of bare NF was calculated. The corresponding GCD profile (at the current density of 1 A g^−1^) is shown in Figure S9. The maximum *Q_s_* for NF was found to be 20.5 C g^−1^ (5.7 mAh g^−1^), which indicates a negligible contribution of NF to the electrochemical performance of the electrodes.

## 4. Conclusions

A unique 3D ternary nanohybrid based on Ni_3_S_2_, CoMoS_4_, and MnO_2_ was synthesized through a simple, facile route and exhibited superior electrochemical properties as an advanced binder-free SC electrode. Strong synergy between the individual components enhanced the electrochemical performance of the ternary binder-free electrode. A solid-state asymmetric SC device based on this ternary nanohybrid provided a moderate ED of 13.8 Wh kg^−1^. In contrast, the aqueous asymmetric device delivered a high ED of 20.7 Wh kg^−1^. Overall, this study explored the smart design of a promising SC electrode and an in-depth understanding of the structure/property relationship.

## Figures and Tables

**Table 1:** Comparison of electrochemical performances of NCMSM//rGO SASC and AASC devices.

Performance Criteria	NCMSM//rGO SASC	NCMSM//rGO AASC
Working potential window	1.4 V	1.6 V
Specific capacitance/capacity (at 1 A g^−1^)	51 F g^−1^/19.2 mAh g^−1^	58.3 F g^−1^/25.1 mAh g^−1^
Maximum ED	13.8 Wh kg^−1^	20.7 Wh kg^−1^
Cycling stability (2000 cycles)	93%	96%

## Data Availability

The data presented in this study are available in the lab research notebook in Yeungnam University.

## Supplementary Materials

The following are available online at https://www.mdpi.com/article/10.3390/nano12111945/s1, synthesis of reduced graphene oxide coated Ni foam (rGO@Ni foam), Equations used to calculate specific capacitance, ED, and PD of the electrode materials, Figure S1. Morphology of the bare Ni foam and NS: SEM images at low and high magnifications for (a,b) bare Ni foam and (c,d) NS., Figure S2. (a) SAED image of NCMSM confirming the polycrystalline nature of MnO_2_, (b) additional TEM image of NCMSM showing ultrathin nanoflakes of MnO_2_., Figure S3. (a) XPS survey analysis of NS confirming the presence of Ni 2p and S 2p levels and (b) cyclic voltammograms of NS at various scan rates ranging from 5 to 100 mV s^−1^ in 1 M KOH, Figure S4. N_2_ adsorption-desorption curves and pore size distributions of (a,b) NCMS and (c,d) NCMSM, Electrochemical Activity of CoMoS_4_ on carbon cloth, Figure S5. SEM images of (a) the bare carbon cloth (CC) and (b) CMS@CC, (c) XRD patterns of CC and CMS@CC, and (d) CV curves for CMS@CC and bare CC at a scan rate of 100 mV s^−1^ in 1 M KOH electrolyte, Figure S6. Nyquist plots of the bare CC and CMS@CC (inset: magnified view of the high-frequency region), Figure S7. (a,b) Low- and high-magnification SEM images of rGO@Ni foam, Figure S8. (a) CV curves at a scan rate of 100 mV s^−1^ of NCMSM//rGO SASC within different potential windows; (b) Galvanostatic charge-discharge profiles of NCMSM//rGO SASC at a current density of 2 A g^−1^ within different potential windows; (c) CV curves at a scan rate of 100 mV s^−1^ for NCMSM//rGO AASC within different potential windows; (d) Galvanostatic charge-discharge profiles of NCMSM//rGO AASC at a current density of 2 A g^−1^ within different potential windows, Figure S9. Galvanostatic charge-discharge profile of bare Ni foam at a current density of 1 A g^−1^ within the potential window of 0–0.45 V, Table S1. Specific capacitances of NCMSM with other reported MnO_2_-based composites, Table S2. Cycling stability values of NCMSM with other reported MnO_2_-based composites, Table S3. Specific capacitances of NCMSM//rGO SASC with other reported asymmetric supercapacitors, Table S4. Specific capacitances of NCMSM//rGO AASC with other reported aqueous asymmetric supercapacitors.
