# Supplementary material for: Ternary Nanohybrid of Ni3S2/CoMoS4/MnO2 on Nickel Foam for Aqueous and Solid-State High-Performance Supercapacitors
Source: Nanomaterials (Basel). 2022 Jun 6;12(11):1945. doi: 10.3390/nano12111945 (PMC9182481; doi:10.3390/nano12111945)
Supplement: Supplementary file 1 [file nanomaterials-12-01945-s001.zip › nanomaterials-1699190-supplementary.pdf]

nanomaterials

MDPI

Supplementary Materials

Ternary Nanohybrid of Ni_3S_2/$CoMoS_4$/MnO_2 on Nickel Foam for Aqueous and Solid-State High-Performance Supercapacitors

Sumanta Sahoo, Ganesh Dhakal, Woo Kyoung Kim, and Jae-Jin Shim *

School of Chemical Engineering, Yeungnam University, 280 Daehak-Ro, Gyeongsan, Gyeongbuk 38541, Korea; sumanta95@gmail.com (S.S.); gdhakal17@gmail.com (G.D.); wkim@ynu.ac.kr (W.K.K.)

* Correspondence: jjshim@yu.ac.kr; Tel.: +82-53-810-2587

S1. Synthesis of Reduced Graphene Oxide Coated Ni Foam (rGO@Ni Foam)

To synthesize rGO@Ni foam for the negative electrode of the asymmetric device, first graphene oxide (GO) powder was prepared by the modified Hummers' method, which is described in one of our previous reports [1]. GO suspension at a 3 mg mL^{-1} concentration was prepared by dispersing GO in DI water through stirring and hydrothermally heated at 180 °C for 6 h to get rGO hydrogel. Lastly, the requisite amount of rGO hydrogel was polished on Ni foam without any binder and dried to obtain rGO@Ni foam.

S2. Equations Used for the Calculation of Specific Capacitance, Energy Density, and Power Density of the Electrode Materials

As the electrode materials displayed non-linear discharge profiles, the following equation was used to calculate the specific capacitance (C_s), where I is the constant current (A); m is the mass of active material (g); ΔV is the discharge potential window (V), and ∫Vdt represents the area under the discharge curve:

$$C_s = \frac{2I\int Vdt}{m(\Delta V)^2} \quad \text{(S1)}$$

The specific capacity (Q_s) was calculated by the following equation:

$$Q_s = \frac{2I\int Vdt}{m(\Delta V)} \quad \text{(S2)}$$

The coulombic efficiency of the electrode materials was calculated by the following equation:

$$\eta = \frac{t_d}{t_c} \quad \text{(S3)}$$

where t_d and t_c are discharge time and charge time, respectively.

The mass ratio of the positive and negative electrodes was calculated by using the following equation to achieve the charge balance between two electrodes as follows:

$$\frac{m_+}{m_-} = \frac{C_- V_-}{C_+ V_+} \quad \text{(S4)}$$

where m_+, m_-, C_+, C_-, V_+, V_- are the mass (g), specific capacitance (measured in a three-electrode system) (F g^{-1}), and potential windows (V) of the positive (+) and negative (−) electrodes. The energy density (ED, Wh kg^{-1}) and power density (PD, W kg^{-1}) were calculated using the following equations:

$$E_D = \frac{I \int V\, dt}{3.6\ \mathrm{M}} \tag{S5}$$

$$P_D = \frac{3600\ E_D}{t} \tag{S6}$$

where t is the discharge time (s), and M is the total mass of the active materials (g) of both electrodes.

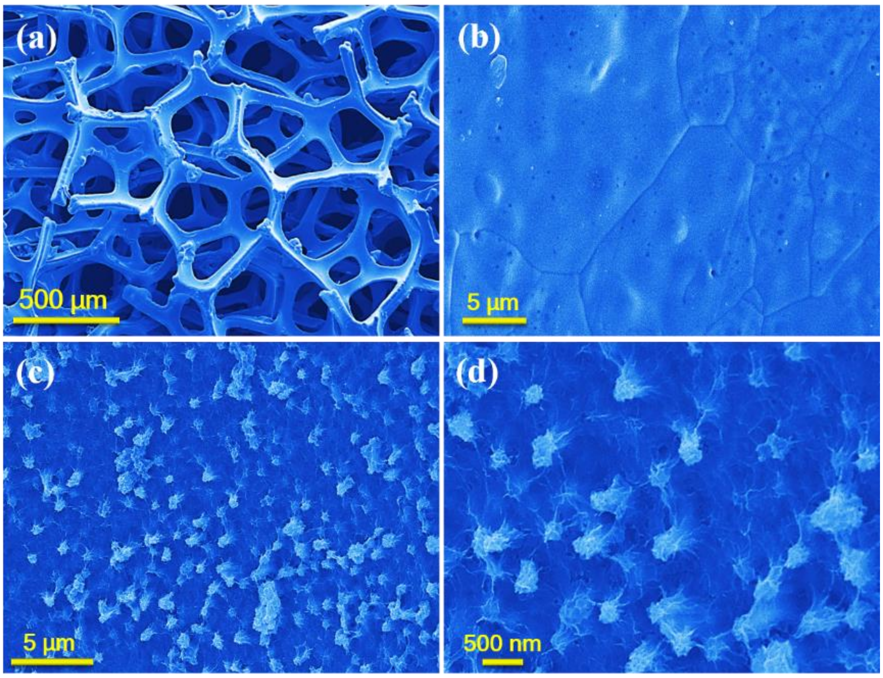

Figure S1. Morphology of the bare Ni foam and NS: SEM images at low and high magnifications for (**a**,**b**) bare Ni foam and (**c**,**d**) NS.

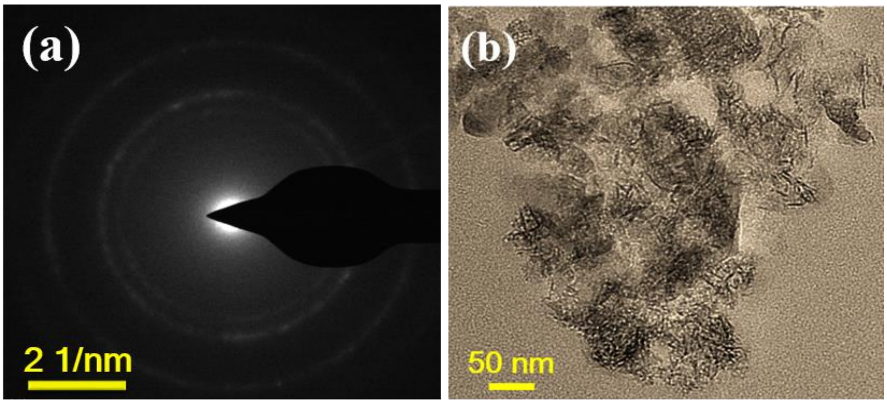

Figure S2. (**a**) SAED image of NCMSM confirming the polycrystalline nature of MnO_2, (**b**) additional TEM image of NCMSM showing ultrathin nanoflakes of MnO_2.

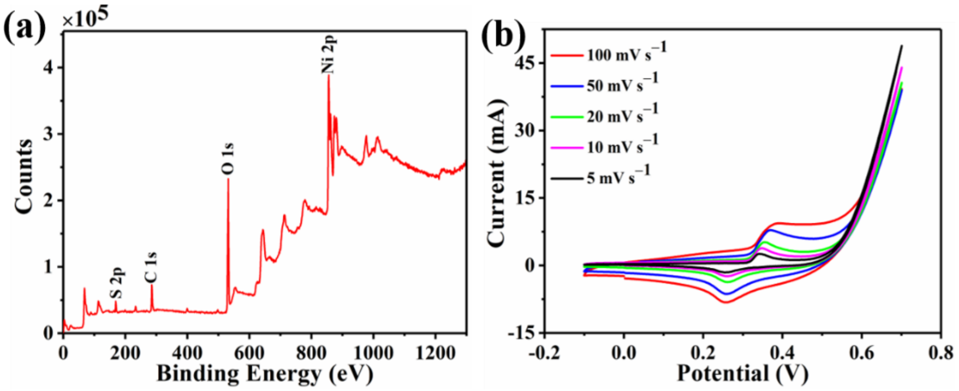

Figure S3. (**a**) XPS survey analysis of NS confirming the presence of Ni 2p and S 2p levels and (**b**) cyclic voltammograms of NS at various scan rates ranging from 5 to 100 mV s^{-1} in 1 M KOH.

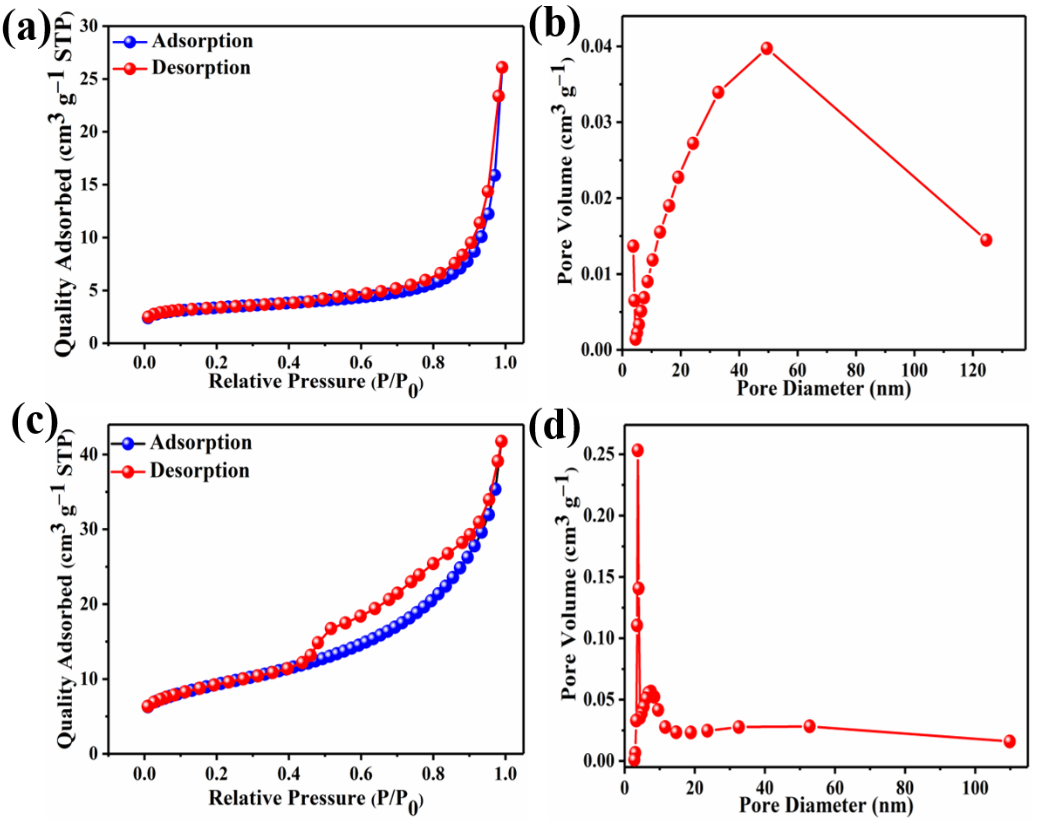

Figure S4. N_2 adsorption-desorption curves and pore size distributions of (**a**,**b**) NCMS and (**c**,**d**) NCMSM.

S3. Electrochemical Activity of $CoMoS_4$ on Carbon Cloth

To check the electrochemical activity of $CoMoS_4$ without the interference of Ni_3S_2, $CoMoS_4$ was grown on a carbon cloth (CC) substrate following similar reaction conditions. The morphological analysis found that the shape of $CoMoS_4$ had changed after using CC as the substrate instead of Ni foam. This phenomenon can be explained by the different template effects of the substrates. As shown in Figure S5b, long nanowires of $CoMoS_4$ were grown on the smooth surface of CC. The lengths of the wires were several micrometers, whereas the thicknesses of the wires ranged from 5–10 nm. This type of nanowire morphology is highly favorable for easy electrolyte transport and hence highly feasible for enhanced electrochemical properties. Figure S5c represents the XRD patterns of CC and CMS@CC. Two prominent peaks at $2\theta = 25.4°$ and $43.4°$ correspond to the (002) and (101) planes of carbon cloth. Except for these two prominent peaks of CC, no noticeable

peaks were observed in the XRD pattern of CMS@CC, which is probably due to the low growth rate of $CoMoS_4$ on CC, the presence of strong peaks of the substrate (CC), and the amorphous nature of CMS. The electrochemical activity of CMS@CC was found to be higher than the substrate CC, as confirmed by the CV curves of CC and CMS@CC (Figure S5d). As compared to the bare CC, the response current for CMS@CC was much higher at the scan rate of 100 mV s^{-1} in the potential window −0.1 to 0.7 V. Additionally, the Nyquist plot of CMS@CC also showed very low impedance as compared to the CC substrate, ensuring high electrical conductivity of $CoMoS_4$ (Figure S6). CMS@CC exhibited a lower ESR value (1.7 Ω) than the bare CC (2.87 Ω). Therefore, we can conclude that $CoMoS_4$ is a superior candidate for supercapacitor applications.

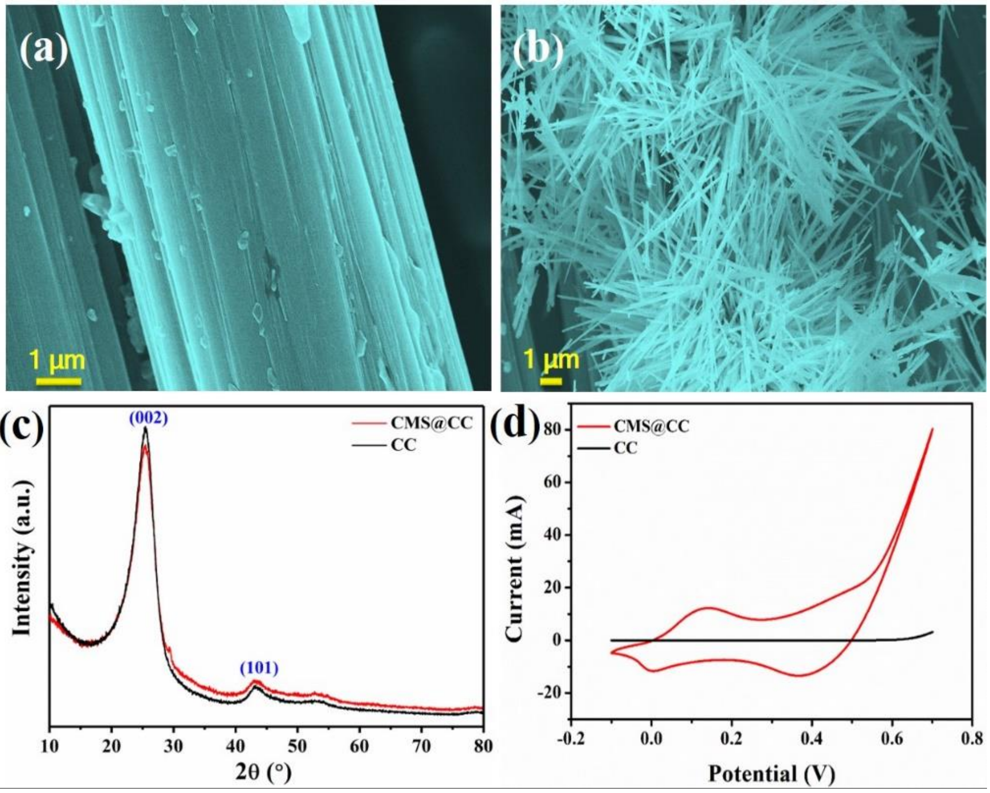

Figure S5. SEM images of (**a**) the bare carbon cloth (CC) and (**b**) CMS@CC, (**c**) XRD patterns of CC and CMS@CC, and (**d**) CV curves for CMS@CC and bare CC at a scan rate of 100 mV s^{-1} in 1 M KOH electrolyte.

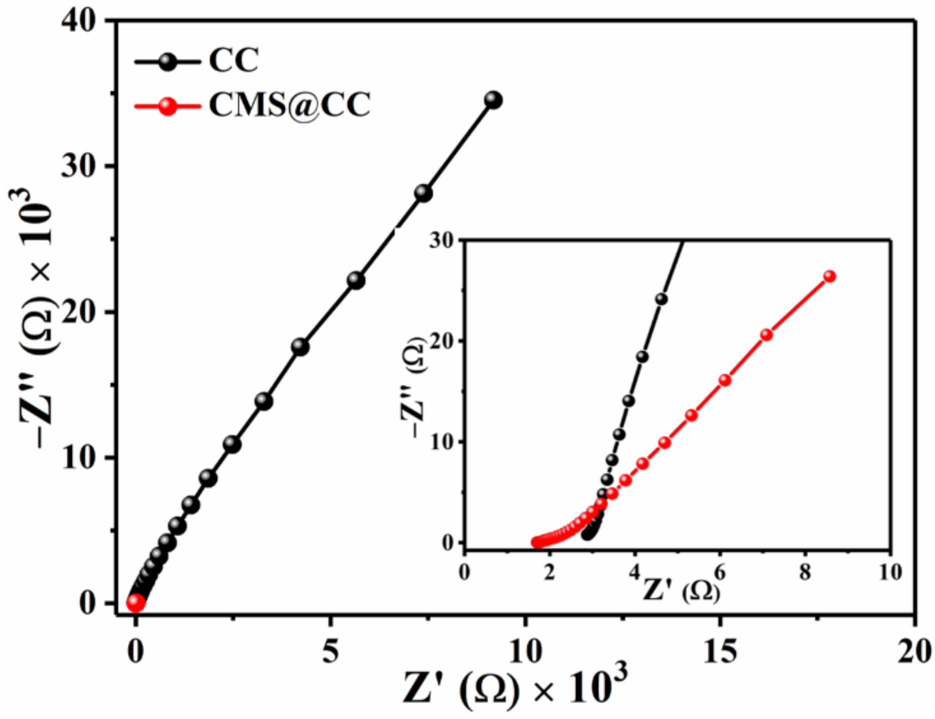

Figure S6. Nyquist plots of the bare CC and CMS@CC (inset: magnified view of the high-frequency region).

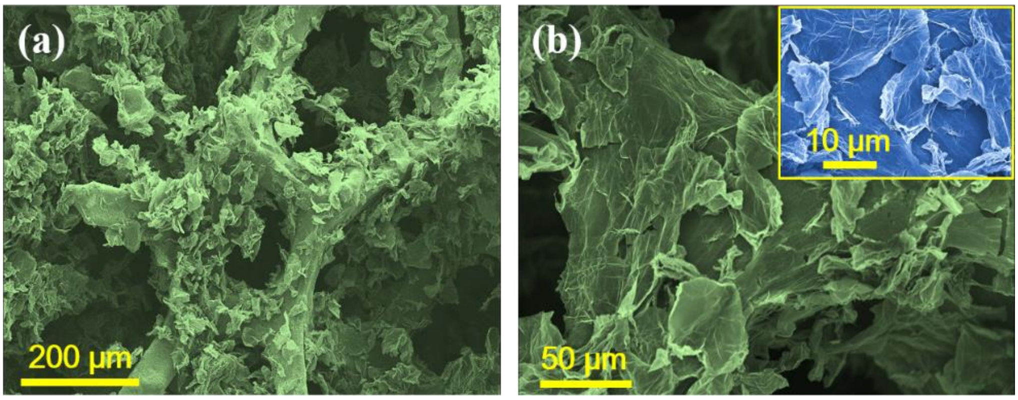

Figure S7. (**a**,**b**) Low and high magnification SEM images of rGO@Ni foam.

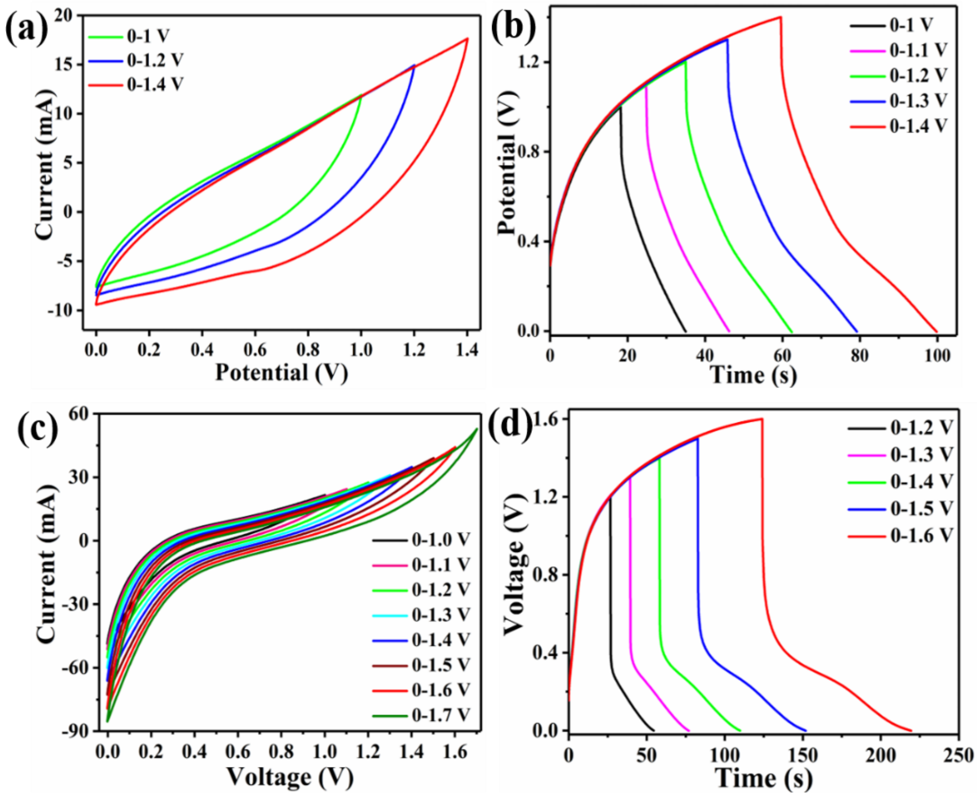

Figure S8. (**a**) CV curves at a scan rate of 100 mV s^{-1} of NCMSM//rGO SASC within different potential windows; (**b**) Galvanostatic charge-discharge profiles of NCMSM//rGO SASC at a current density of 2 A g^{-1} within different potential windows; (**c**) CV curves at a scan rate of 100 mV s^{-1} for NCMSM//rGO AASC within different potential windows; (**d**) Galvanostatic charge-discharge profiles of NCMSM//rGO AASC at a current density of 2 A g^{-1} within different potential windows.

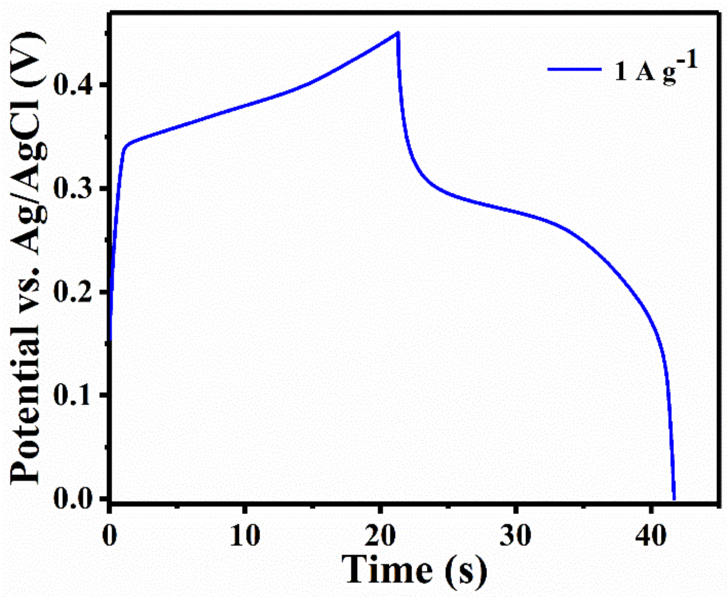

Figure S9. Galvanostatic charge-discharge profile of bare Ni foam at a current density of 1 A g^{-1} within the potential window of 0–0.45 V.

Table S1. Specific capacitances of NCMSM with other reported MnO_2-based composites.

Serial No.	Supercapacitor Electrode Materials	Specific Capacitance (F g⁻¹)	Test Condition	References
1	MnO_2@$NiCo_2S_4$	520.7	1 A g^{-1}	[2]
2	MnO_2@Co_3O_4	560	0.2 A g^{-1}	[3]
3	MnO_2 grafted V_2O_5	450	0.5 A g^{-1}	[4]
4	MnO_2@ZnO	423.5	0.5 A g^{-1}	[5]
5	MnO_2@Co_3O_4 nanoconch arrays	1183.7	1 A g^{-1}	[6]
6	SnO_2@MnO_2	367.5	50 mV g^{-1}	[7]
7	MnO_2@$CuCo_2O_4$	416	1 A g^{-1}	[8]
8	MnO_2@$MnCo_2O_4$	858	1 A g^{-1}	[9]
9	MnO_2@CuO	228	0.25 A g^{-1}	[10]
**10**	**NCMSM**	**2021**	**1 A g^{-1}**	**This work**

Table S2. Cycling stability values of NCMSM with other reported MnO_2-based composites.

Serial No.	Supercapacitor Electrode Materials	Cycling Stability (%)	Number of Cycles	References
1	MnO_2@$NiCo_2O_4$	88	2000	[11]
2	MnO_2@$NiCo_2S_4$	82	2000	[12]
3	MnO_2@$NiMoO_4$	74.8	3000	[13]
4	MnO_2 grafted V_2O_5	89	500	[4]
5	MnO_2@NiO	81.7	2000	[14]
6	MnO_2@RuO_2	80	1000	[15]
**7**	**NCMSM**	**90**	**4000**	**This work**

Table S3. Specific capacitances of NCMSM//rGO SASC with other reported asymmetric supercapacitors.

Serial No.	Asymmetric Supercapacitors	Specific Capacitance (F g⁻¹)	Test Condition	References
1	Fe_3O_4//AC	37.9	0.5 mA cm^{-1}	[16]
2	CoS//AC	47	2 A g^{-1}	[17]
3	$Co(OH)_2$/graphene//AC	50	1 A g^{-1}	[18]
**4**	**NCMSM//rGO SASC**	**51**	**1 A g^{-1}**	**This work**

AC: Activated Carbon; SASC: Solid-state asymmetric supercapacitor.

Table S4. Specific capacitances of NCMSM//rGO AASC with other reported aqueous asymmetric supercapacitors.

Serial No.	Asymmetric Supercapacitors	Specific capacitance (F g⁻¹)	Test condition	References
1	PNPG/Au/SS//AC	38	0.5 A g^{-1}	[19]
2	MnO_2//AC	30	1 mV s^{-1}	[20]
3	CoMn-LDH//AC	24.2	0.5 A g^{-1}	[21]
4	PPy/PMA//PEDOT/PTA	31	1 mA	[22]
**5**	**NCMSM//rGO AASC**	**58.3**	**1 A g^{-1}**	**This work**

PNPG: Poly(N-phenylglycine); SS: Stainless Steel; PPy: Polypyrrole; PMA: Phosphomolybdic Acid; PEDOT: Poly(3,4-ethylenedioxythiophene); PTA: Phosphotungstic Acid; AASC: Aqueous asymmetric supercapacitor.

References

1. Sahoo, S.; Zhang, S.; Shim, J.-J. Porous Ternary High Performance Supercapacitor Electrode Based on Reduced Graphene Oxide, $NiMn_2O_4$, and Polyaniline. *Electrochim. Acta* **2016**, *216*, 386–396, https://doi.org/10.1016/j.electacta.2016.09.030.
2. Chen, H.; Liu, X.L.; Zhang, J.M.; Dong, F.; Zhang, Y.X. Rational synthesis of hybrid $NiCo_2S_4$@MnO_2 heterostructures for supercapacitor electrodes. *Ceram. Int.* **2016**, *42*, 8909–8914, https://doi.org/10.1016/j.ceramint.2016.02.146.
3. Huang, M.; Zhang, Y.; Li, F.; Zhang, L.; Wen, Z.; Liu, Q. Facile synthesis of hierarchical Co_3O_4@MnO_2 core–shell arrays on Ni foam for asymmetric supercapacitors. *J. Power Sources* **2014**, *252*, 98–106, https://doi.org/10.1016/j.jpowsour.2013.12.030.
4. Saravanakumar, B.; Purushothaman, K.K.; Muralidharan, G. MnO_2 grafted V_2O_5 nanostructures: formation mechanism, morphology and supercapacitive features. *CrystEngComm* **2014**, *16*, 10711–10720.
5. Huang, M.; Li, F.; Zhao, X.L.; Luo, D.; You, XQ.; Zhang, Y.X.; Li, G. Hierarchical ZnO@ MnO_2 core-shell pillar arrays on Ni foam for binder-free supercapacitor electrodes. *Electrochim. Acta* **2015**, *152*, 172–177.
6. Qiu, K.; Yan, H.; Zhang, D.; Lu, Y.; Cheng, J.; Lu, M.; Wang, C.; Zhang, Y.; Liu, X.; Luo, Y. Hierarchical 3D Co_3O_4@MnO_2 core/shell nanoconch arrays on Ni foam for enhanced electrochemical performance. *J. Solid State Electrochem.* **2014**, *19*, 391–401, https://doi.org/10.1007/s10008-014-2611-z.
7. Dai, Y.; Tang, S.; Peng, J.; Chen, H.; Ba, Z.; Ma, Y.; Meng, X. MnO_2@SnO_2 core–shell heterostructured nanorods for supercapacitors. *Mater. Lett.* **2014**, *130*, 107–110, https://doi.org/10.1016/j.matlet.2014.05.090.
8. Kuang, M.; Liu, X.Y.; Dong, F.; Zhang, Y.X. Tunable design of layered $CuCo_2O_4$ nanosheets@MnO_2 nanoflakes core–shell arrays on Ni foam for high-performance supercapacitors. *J. Mater. Chem. A* **2015**, *3*, 21528–21536.
9. Zheng, X.; Ye, Y.; Yang, Q.; Geng, B.; Zhang, X. Hierarchical structures composed of $MnCo_2O_4$@MnO_2 core–shell nanowire arrays with enhanced supercapacitor properties. Dalton Transactions **2016**, *45*, 572–578.
10. Zhang, Z.; Ma, C.; Huang, M.; Li, F.; Zhu, S.; Hua, C.; Yu, L.; Zheng, H.; Hu, X.; Zhang, Y. Birnessite MnO_2-decorated hollow dandelion-like CuO architectures for supercapacitor electrodes. *J. Mater. Sci. Mater. Electron.* **2015**, *26*, 4212–4220, https://doi.org/10.1007/s10854-015-2969-4.
11. Yu, L.; Zhang, G.; Yuan, C.; Lou, X.W.D. Hierarchical $NiCo_2O_4$@MnO_2 core-shell heterostructured nanowire arrays on Ni foam as high-performance supercapacitor electrodes. *Chem. Commun.* **2013**, *49*, 137–139.
12. Yang, J.; Ma, M.; Sun, C.; Zhang, Y.; Huang, W.; Dong, X. Hybrid $NiCo_2S_4$@MnO_2 heterostructures for high-performance supercapacitor electrodes. *J. Mater. Chem. A* **2015**, *3*, 1258–1264.
13. Ma, X.-J.; Zhang, W.-B.; Kong, L.-B.; Luo, Y.-C.; Kang, L. $NiMoO_4$-modified MnO_2 hybrid nanostructures on nickel foam: electrochemical performance and supercapacitor applications. *New J. Chem.* **2015**, *39*, 6207–6215, https://doi.org/10.1039/C5NJ00639B.
14. Chen, J.; Huang, Y.; Li, C.; Chen, X.; Zhang, X. Synthesis of NiO@MnO_2 core/shell nanocomposites for supercapacitor application. *Appl. Surf. Sci.* **2016**, *360*, 534–539.
15. Chou, J.-C.; Chen, Y.-L.; Yang, M.-H.; Chen, Y.-Z.; Lai, C.-C.; Chiu, H.-T.; Lee, C.-Y.; Chueh, Y.-L.; Gan, J.-Y. RuO_2/MnO_2 core-shell nanorods for supercapacitors. *J. Mater. Chem. A* **2013**, *1*, 8753–8758.
16. Du, X.; Wang, C.; Chen, M.; Jiao, Y.; Wang, J. Electrochemical performances of nanoparticle Fe_3O_4/activated carbon supercapacitor using KOH electrolyte solution. *J. Phys. Chem. C* **2009**, *113*, 2643–2646.
17. Subramani, K.; Sudhan, N.; Divya, R.; Sathish, M. All-solid-state asymmetric supercapacitors based on cobalt hexacyanoferrate-derived CoS and activated carbon. *RSC Adv.* **2017**, *7*, 6648–6659, https://doi.org/10.1039/c6ra27331a.
18. Zhao, C.; Ren, F.; Xue, X.; Zheng, W.; Wang, X.; Chang, L. A high-performance asymmetric supercapacitor based on $Co(OH)_2$/graphene and activated carbon electrodes. *J. Electroanal. Chem.* **2016**, *782*, 98–102.
19. Li, M.; Luo, Y.; Jia, C.; Huang, M.; Yu, M.; Luo, G.; Zhao, L.; Boukherroub, R.; Jiang, Z. Au-assisted polymerization of conductive poly(N-phenylglycine) as high-performance positive electrodes for asymmetric supercapacitors. *Nanotechnology* **2021**, *33*, 045602, https://doi.org/10.1088/1361-6528/ac1fb3.
20. Liu, H.; Zhao, K. Asymmetric flow electrochemical capacitor with high energy densities based on birnessite-type manganese oxide nanosheets and activated carbon slurries. *J. Mater. Sci.* **2016**, *51*, 9306–9313, https://doi.org/10.1007/s10853-016-0177-0.
21. Jagadale, A.; Guan, G.; Li, X.; Du, X.; Ma, X.; Hao, X.; Abudula, A. Ultrathin nanoflakes of cobalt–manganese layered double hydroxide with high reversibility for asymmetric supercapacitor. *J. Power Sources* **2016**, *306*, 526–534, https://doi.org/10.1016/j.jpowsour.2015.12.097.
22. Suppes, G.M.; Cameron, C.G.; Freund, M.S. A Polypyrrole/Phosphomolybdic Acid/Poly(3,4-ethylenedioxythiophene)/Phosphotungstic Acid Asymmetric Supercapacitor. *J. Electrochem. Soc.* **2010**, *157*, A1030–A1034, https://doi.org/10.1149/1.3464802.
